# Current practices of peripheral intravenous catheter fixation in pediatric patients and factors influencing pediatric nurses’ knowledge, attitude and practice concerning peripheral intravenous catheter fixation: a cross-sectional study

**DOI:** 10.1186/s12912-021-00758-1

**Published:** 2021-11-23

**Authors:** Li-Sha Huang, Yan Huang, Juan Hu

**Affiliations:** 1 Department of Emergency Nursing, West China Second University Hospital, Sichuan University/West China School of Nursing, Sichuan University; Key Laboratory of Birth Defects and Related Diseases of Women and Children (Sichuan University), Ministry of Education, Chengdu, Sichuan China; 2 Department of Nursing, West China Second University Hospital, Sichuan University/West China School of Nursing, Sichuan University; Key Laboratory of Birth Defects and Related Deceases of Woman and Children (Sichuan University), Ministry of Education, Chengdu, Sichuan China

**Keywords:** Pediatric nurses, Peripheral intravenous catheter, Fixation, Knowledge, Attitude, Practice

## Abstract

**Background:**

Peripheral intravenous catheters (PIVCs) are the most widely used intravenous treatment tools for hospitalized patients. Compared to adult patients, PIVC fixation issues are more likely to occur in pediatric patients and can be more complex. However, research on PIVC fixation in pediatric patients is rare. This study aimed to investigate the pass rate for PIVC fixation in pediatric patients and the factors that influence pediatric nurses’ knowledge, attitude, and practice (KAP) concerning PIVC fixation.

**Methods:**

An on-site investigation using a self-designed PIVC fixation standard inspection checklist for first insertion and routine maintenance in pediatric patients and a follow-up questionnaire survey investigating pediatric nurses’ KAP concerning PIVC fixation was conducted in a hospital in China between November 1 and December 31, 2019. Data were analyzed using SPSS 21.0.

**Results:**

The pass rate for PIVC fixation in pediatric patients was 52.02%. The pediatric nurses’ knowledge, attitude and practice scores on PIVC fixation were 7.2 ± 1.36, 28.03 ± 2.42, and 31.73 ± 2.94, respectively. The multivariate linear regression analysis results show that department (where nurses are working in) and job position are the factors that influence knowledge score (B > 0, *P* < 0.05); department is also a factor that influences attitude score (B > 0, *P* < 0.05); and department and nursing hierarchy are the factors that influence practice score (B > 0, *P* < 0.05).

**Conclusion:**

PIVC fixation in pediatric patients is affected by multiple factors. The level of pediatric nurses’ KAP on PIVC fixation needs to be improved. It is suggested that guidelines for PIVC fixation in pediatric patients be formulated and that training on PIVC fixation in pediatric patients be provided for pediatric nurses in an effort to raise the pass rate in terms of PIVC fixation in pediatric patients.

**Supplementary Information:**

The online version contains supplementary material available at 10.1186/s12912-021-00758-1.

## Background

Peripheral intravenous catheters (PIVCs) are the most widely used intravenous treatment tools for hospitalized patients [1, 2]. Previous studies have shown that PIVCs are administered to approximately 80% of inpatients for fluid supplementation, medication, blood transfusion, etc. [3]. A PIVC can be retained in an adult’s vein for a long period of time (72–96 h). Compared with children, PIVC dwell times are longer in adults, which could prevent potential complications related to multiple cannulations and repeated attempts. However, the safety of using PIVCs depends to a great extent on the quality of PIVC maintenance. PIVC maintenance failures can inflict much pain, increase the risk of infusion leakage, and undermine treatment (particularly when intravenous treatment access is not established quickly). Moreover, compared to adults, the problem of PIVC maintenance is particularly prominent in children [4]. Fixation is a significant procedure in pediatric PIVC maintenance.

The PIVC fixation method normally proceeds as follows: (1) the PIVC and infusion tube are secured with tension-free dressing; (2) the dressing should be shaped based on the shape of the Y joint of the PIVC; (3) the dressing is pressed from the center to the edge; then, (4) using tape, and without applying too much pressure, the root of the extension line is fixed in an omega (Ω) shape.

Regarding PIVC maintenance, fixation problems are more likely to occur among pediatric patients than among adult patients. The average indwelling time with pediatric patients is 29–60 h, which is shorter than that with adult patients; there are many reasons for this difference, for instance, the occurrence of complications. Factors explaining such complications include pediatric patients’ physical factors, e.g., hyperactivity and perspiration [5]. However, tight fixation may cause discomfort, thereby affecting blood circulation and iatrogenic skin injury, while loose fixation may lead to accidental slippage of the PIVC or infection [6, 7]. Poor-quality PIVC fixation can lead to unplanned removal and pressure-induced skin injury [8]. For these reasons alone, it is essential for pediatric nurses to master standard methods of PIVC fixation for the sake of patient safety during intravenous therapy. In addition, based on knowledge, attitude, and practice (KAP) as the theoretical framework, the appropriate KAP [9] levels of PIVC fixation among pediatric nurses who are responsible for fixing PIVCs are critical to the quality of PIVC fixation in pediatric patients. PIVC fixation in pediatric patients may be more complex than that in adult patients. The quality of PIVC fixation directly affects the service life of the PIVC, as well as patient safety. However, most previous PIVC studies and guidelines have focused on adult patients; there are relatively few PIVC studies that are focused on pediatric patients, and these are focused mainly on PIVC insertion, bloodstream-associated infection, and flushing [10–13], while studies on fixation in pediatric patients are rare. We aimed to investigate the pass rate for PIVC fixation in pediatric patients and factors influencing pediatric nurses’ KAP concerning peripheral intravenous catheter fixation.

## Methods

### Study setting

An on-site investigation using a self-designed PIVC fixation standard inspection checklist for first puncture and routine maintenance in pediatric patients and a follow-up questionnaire survey investigating pediatric nurses’ KAP of PIVC fixation were conducted in the West China Second University Hospital, Sichuan University between November 1 and December 31, 2019.

### Research objects and participants

The on-site investigation was conducted only on PIVCs that were implanted and maintained by nurses from the pediatric emergency department, department of general pediatrics, and pediatric intensive care unit at the West China Second University Hospital, Sichuan University. PIVCs that were implanted at other hospitals were excluded from this study.

The questionnaire survey was conducted by a selected sample of the aforementioned nurses. The inclusion criteria were as follows: (1) voluntarily participation in this study; (2) registered nurses; (3) nurses who worked in the pediatric emergency department, department of general pediatrics, or pediatric intensive care unit. Trainee nurses and logistic nurses were excluded from this study.

### Study tools

A self-designed PIVC fixation standard inspection checklist (Supplement 1) was used to investigate the PIVC fixation pass rate among the enrolled pediatric nurses. The checklist was composed of two parts; Part 1 concerned the first puncture, and Part 2 concerned routine maintenance. Each of the two parts contained 3 components, namely, bed number (1 question), checklist items (5 questions), and results (1 question). Both parts contained the same 5 questions in checklist items, namely, “Was the dressing fixed with the standard technique?”, “Was the needle joint fixed with the Ω technique?”, “Were auxiliary fixtures used correctly?”, and “Was health education provided?” Both parts contained the same question about the result, namely, “Was the PIVC fixation rated as ‘pass’?” The remaining questions in the checklist items in Parts 1 and 2 were as follows: “Did the skin dry naturally after performing skin disinfection?” and “Was the dressing replaced in time when it was abnormal?” The investigators placed a “√” in the answer box for “Yes” and an “×” for “No”. PIVC fixation techniques were rated “pass” only if all 5 questions in the checklist items were marked with a “√”. The whole inspection process was witnessed by the head nurse of the participant’s respective department. On-site investigators took photographs demonstrating the PIVC fixation procedure while filling in the checklist.

The self-designed questionnaire (Supplement 2) based on the infusion therapy standards of practice of the Infusion Nurses Society [13] and the KAP framework was composed of two parts. Part 1 was composed of 7 questions; the respondents were asked to state which department they were working in, their years of experience, their professional title, their position in the nursing hierarchy, their educational background, their job position, and whether they had received intravenous therapy training.

Part 2 was composed of 23 questions, among which 10 concerned knowledge, 6 concerned attitude, and 7 concerned practice. Three response options, namely, “right”, “wrong”, and “don’t know”, were provided for the 10 questions concerning knowledge. A score of 1 was assigned to answers of “right”, and a score of 0 was assigned to answers of “wrong” and “don’t know”. A 5-point Likert scale was used for questions about attitude and practice. For the 6 questions concerning attitude, scores ranged from 1 to 5 for “not important at all”, “not important”, “moderately important”, “important”, and “very important”. For the 7 questions concerning practice, scores ranged from 1 to 5 for “never”, “occasionally”, “sometimes”, “often”, and “always”. A higher KAP score indicated a higher KAP level. The standard score = (actual score/full score) × 100. This study defined a standard score of < 60 as poor, 60–80 as moderate, and > 80 as good.

### Data collection

Data were collected using a convenience cluster sampling approach. A QR code linking to the questionnaire was shared with approximately 600 pediatric nurses working at the hospital using a WeChat group chat by a member of the Intravenous Treatment Nursing Workshop (i.e., the head nurse of the emergency department, the corresponding author of this study, Juan Hu). Every nurse participated in this study voluntarily by scanning the QR code and completing the questionnaire. There were some restrictions in the design of this electronic questionnaire to ensure data validity. For example, each WeChat account could only log in and fill in the questionnaire once, and the time limit was 15 min. At the beginning of the questionnaire, participants were required to check whether they were pediatric nurses and registered nurses to ensure that each participant met the inclusion criteria.

To estimate the rate of accurate PIVC fixation, we calculated the minimum sample size by using the following formula:

$$ \mathrm{n}=\frac{{\mathrm{u}}_{\alpha /2}^2\mathrm{p}\left(1-\mathrm{p}\right)}{\delta^2} $$

In our study, α = 0.05, the tolerance error (δ) was 0.033, and the rate of accurate PIVC fixation (p) was 79% [14], as reported in a previous study. According to the above parameters, the minimum sample size (n) was 585. Considering a failure rate of 10%, we increased the sample size to 650. In this study, a total of 650 PIVC fixation cases were inspected, of which 642 cases were valid (valid inspection rate = 98.78%).

To estimate pediatric nurses’ KAP level of PIVC fixation,we calculated the minimum sample size of PIVC fixation questionnaire by the recommendation of the sample size described in Determining Sample Size for Research Activities, approximately 600 pediatric nurses met the participant inclusion criteria of this study, and the recommended minimum sample size of our study was 234 pediatric nurses. In this study, a total of 450 questionnaires were collected, of which 433 questionnaires were valid and 17 were incomplete (valid recovery rate = 96.22%).

### Statistical analysis

The data were analyzed using SPSS 21.0. Numerical data are described with reference to the sample size, i.e. number of cases (n). Measurement data that had a nonnormal distribution in the Shapiro-Wilk normality test are expressed as medians (quartiles).

Comparisons between groups were analyzed using the nonparametric rank-sum test. Multiple factor analysis of KAP scores (treated as dependent variables) was conducted using a multivariate linear regression model. Statistically significant differences identified in single-factor analysis of the independent variable were entered into a multivariate linear regression equation (alpha-to-enter significance level = 0.05, alpha-to-remove significance level = 0.10). The reference group was deemed the dummy variable, and the partial regression coefficient (Beta) was applied.

### Ethics approval

All methods were performed in accordance with the relevant guidelines and regulations. This study was performed in accordance with the Declaration of Helsinki. Ethical approval of this study was obtained from the Medical Ethics Committee of West China Second University Hospital, Sichuan University. Investigators verbally articulated the purpose and significance of this study to pediatric patients and their parents prior to PIVC fixation inspection and informed them that PIVC fixation inspection would commence only with their verbal consent and would cease immediately if they withdrew their consent. For the questionnaire survey, whenever each participant scanned the QR code, they were presented with a statement about the purpose and significance of the study and informed that their participation was voluntary. Respondents who filled in and submitted the questionnaire were regarded as individuals who verbally consented to participate in this study. All the respondents completed the questionnaire anonymously.

## Results

### PIVC fixation standard inspection

Among the 642 valid PIVC fixation cases, 150 cases involved first puncture and 492 cases involved routine maintenance. A total of 85 cases involving first puncture were rated as “pass”, thereby demonstrating a pass rate of 56.67%. A total of 249 cases involving routine maintenance were rated as “pass”, thereby demonstrating a pass rate of 50.61%. The overall pass rate for PIVC fixation in pediatric patients was 52.02%.

### Demographic information of pediatric nurses

As shown in Table 1, among the 433 nurses, 44.34% were from the pediatric intensive care unit, 50.11% had less than 3 years of experience, 40.65% were nurse practitioners (junior professional title), 54.04% were ranked as CN1 or below in the nursing hierarchy, 71.82% possessed an undergraduate qualification, 87.52% were primary nurses, and 87.06% had received intravenous therapy training.

**Table 1:** Demographic information of pediatric nurses and comparison of knowledge, attitude and practice scores on PIVC fixation among nurses with different demographic characteristics [P50 (P25, P75)]

Item	n	%	Knowledge		Attitude		Practice	
			Score	P	Score	P	Score	P
Department				< 0.001		0.044		0 < .001
Pediatric Emergency Department	103	23.78	7.00 (6.00–8.00)		29.00 (25.00–30.00)		31.00 (28.00–32.00)	
Pediatric Intensive Care Unit	192	44.34	8.00 (7.00–8.00) ^a^		30.00 (27.00–30.00)^a^		33.00 (31.00–34.00) ^a^	
Department of General Pediatrics	138	31.87	7.00 (6.00–8.00) ^a^		29.00 (26.00–30.00)		33.00 (30.00–35.00) ^a^	
Years of experience				0.035		0.869		0.052
< 3 years	217	50.11	7.00 (6.00–8.00)		30.00 (26.00–30.00)		32.00 (29.00–34.00)	
3–5 years	43	9.9	7.00 (7.00–8.00)		29.00 (26.00–30.00)		32.00 (31.00–34.00)	
6–10 years	82	18.93	7.00 (6.00–8.00)		29.00 (26.00–30.00)		32.50 (31.00–34.00)	
≥ 11 years	91	21.01	8.00 (7.00–9.00)^a^		29.00 (26.00–30.00)		33.00 (31.00–35.00)	
Professional title				0.093		0.531		0.026
Nurse	165	38.11	7.00 (6.00–8.00)		30.00 (26.00–30.00)		32.00 (29.00–34.00)	
Nurse practitioner	176	40.65	7.00 (6.00–8.00)		29.00 (26.00–30.00)		32.00 (30.00–34.00)	
Supervising nurse	92	21.25	8.00 (6.00–8.75)		29.50 (26.00–30.00)		33.00 (31.00–35.00)^a^	
Nursing hierarchy				0.216		0.494		0.038
CN0	140	32.33	7.00 (6.00–8.00)		29.00 (26.00–30.00)		31.00 (29.00–34.00)	
CN1	94	21.71	7.00 (6.00–8.00)		30.00 (26.00–30.00)		33.00 (30.00–34.00)	
CN2	122	28.18	7.00 (6.00–8.00)		29.00 (25.75–30.00)		32.00 (30.00–34.00)	
CN3	77	17.78	8.00 (6.00–9.00)		30.00 (26.00–30.00)		33.00 (31.00–35.00)^a^	
Educational background				0.418		0.623		0.976
Junior college diploma	104	24.02	7.00 (6.00–8.00)		29.00 (26.00–30.00)		32.50 (30.00–34.00)	
Undergraduate	311	71.82	7.00 (6.00–8.00)		29.00 (26.00–30.00)		32.00 (30.00–34.00)	
Postgraduate	18	4.16	8.00 (6.00–9.00)		29.50 (28.75–30.00)		33.00 (31.00–33.25)	
Intravenous therapy training				0.006		0.750		0.025
Yes	56	87.06	7.00 (6.00–8.00)		29.00 (26.00–30.00)		31.00 (29.00–33.00)	
No	377	12.93	7.00 (6.00–8.00)		29.00 (26.00–30.00)		33.00 (30.00–34.00)	
Job position				0.000		0.677		0.810
Primary nurse	379	87.52	7.00 (6.00–8.00)		29.00 (26.00–30.00)		32.00 (30.00–34.00)	
Nursing manager	31	7.15	8.00 (7.00–9.00)^a^		30.00 (27.00–30.00)		32.00 (28.00–35.00)	
Nursing educator	23	5.31	8.00 (7.00–8.00)		30.00 (26.00–30.00)		32.00 (31.00–34.00)	

Note: *P* < 0.05 meant statistically significant difference. Compared with the first group, ^a^*P* <0.05; Nursing hierarchy: nurses were divided into CN0-CN4 according to their nursing skill levels, years of experience, professional title, and educational background; CN0 - probationary nurse, CN1 - primary nurse, CN2 - qualified nurse, CN3 - professional nurse, and CN4 - clinical nursing expert. In this study, clinical nurse levels are mainly divided into CN0-CN4. CN0 nurse refers to the nurse on probation who was new to the job and has worked for less than 1 year. CN1 nurse refers to the primary nurse who has completed the probation period assessment, has worked for more than 1 year, and can complete general clinical nursing work; CN2 nurse refers to a qualified nurse who has worked as a CN1 nurse for more than 3 years and can complete clinical, teaching and scientific nursing research work. CN3nurse refers to a clinical nursing specialist who has worked as a CN2 nurse for at least 5 years; CN4nurse refers to a clinical management expert who has worked as a CN3 nurse for more than 5 years and holds senior professional titles

### Questionnaire on the KAP of PIVC fixation among pediatric nurses

The average knowledge score was 7.2 ± 1.36, which was converted into a standard score of 72.03 ± 13.57. The proportion of respondents with good standard scores (> 80) on knowledge only accounted for 45.72%. The average score for attitude was 28.03 ± 2.42, which was converted into a standard score of 93.41 ± 8.07. The average score for practice was 31.73 ± 2.94, which was converted into a standard score of 90.66 ± 8.41.

### Comparison of pediatric nurses’ KAP scores on PIVC fixation

As shown in Table 1, statistically significant differences were detected regarding knowledge scores among the nurses in regard to department, years of experience, intravenous therapy training, and job position (*P* < 0.05). Pediatric emergency department nurses’ scores for knowledge were significantly lower than those of pediatric intensive care unit nurses and department of general pediatrics nurses. Furthermore, knowledge scores among nurses with 11 years of experience or above were significantly higher than those among nurses with less than 3 years of experience, while the scores of nurses who received intravenous therapy training were significantly higher than those of nurses who did not receive such training. Finally, the scores of nursing managers were significantly higher than those of primary nurses, while no statistically significant differences were detected in pairwise comparisons among the other groups (*P* > 0.05).

For attitude, a statistically significant difference was detected among the scores of nurses in different departments (*P* < 0.05). Pediatric intensive care unit nurses’ scores were significantly higher than those of pediatric emergency department nurses; however, no statistically significant differences were detected in the scores among the other groups (*P* > 0.05) (Table 1).

For practice, statistically significant differences were detected in the scores among the nurses according to department, professional titles, nursing hierarchy, and intravenous therapy training (*P* < 0.05). Pairwise comparisons between groups showed that the scores of pediatric intensive care unit nurses and the department of general pediatrics nurses were significantly higher than those of pediatric emergency department nurses. The scores of supervising nurses were significantly higher than those of ordinary nurses. The scores of nurses ranked as CN3 in the hierarchy were significantly higher than those of nurses ranked as CN0. The scores of nurses who received intravenous therapy training were significantly higher than those of nurses who did not receive such training. No statistically significant differences were detected among the practice scores of nurses for other factors (*P* > 0.05) (Table 1).

### Multiple linear regression analysis of a questionnaire investigating pediatric nurses’ KAP concerning PIVC fixation

The multivariate linear regression analysis results show that department (where nurses are working in) and job position are the factors that influence knowledge score (B > 0, *P* < 0.05); department is also a factor that influences attitude score (B > 0, *P* < 0.05); and department and nursing hierarchy are the factors that influence practice score (B > 0, *P* < 0.05) (Table 2).

**Table 2:** Multivariate linear regression analysis on factors influencing pediatric nurses’ knowledge, attitude and practice of PIVC fixation

Dependent variable	Independent variable	Partial regression coefficient (B)	Standard error (SE)	Standardized regression coefficient (b)	t	*P*	95% CI	
							Lower limit	Upper limit
Knowledge	**Constant (quantity)**	6.199	0.207		29.935	< 0.001	5.792	6.606
	Department (Take Pediatric Emergency Department as a reference)							
	Pediatric Intensive Care Unit	0.794	0.159	0.291	4.986	< 0.001	0.481	1.107
	Department of General Pediatrics	0.544	0.170	0.187	3.195	0.002	0.209	0.879
	Job position (Take primary nurse as a reference)							
	Nursing manager	0.768	0.257	0.146	2.990	0.003	0.263	1.272
	Nursing educator	0.462	0.291	0.076	1.592	0.112	− 0.109	1.033
	Intravenous therapy training	0.352	0.190	0.087	1.856	0.064	−0.021	0.725
Attitude	Constant (quantity)	27.583	0.443		62.300	< 0.001	26.713	28.453
	Department (Take Pediatric Emergency Department as a reference)							
	Pediatrics Intensive Care Unit	0.692	0.300	0.142	2.305	0.022	0.102	1.281
	Department of General Pediatrics	0.171	0.321	0.033	0.533	0.595	−0.461	0.803
Practice	**Constant (quantity)**	29.328	0.474		61.830	< 0.001	28.396	30.260
	Department (Take Pediatric Emergency Department as a reference)							
	Pediatric Intensive Care Unit	1.874	0.346	0.316	5.412	< 0.001	1.194	2.555
	Department of General Pediatrics	1.501	0.372	0.238	4.039	< 0.001	0.771	2.232
	Nursing hierarchy (Take CN0 as a reference)							
	CN1	1.123	0.558	0.157	2.013	0.045	0.027	2.220
	CN2	1.286	0.675	0.196	1.905	0.057	−0.041	2.613
	CN3	0.759	1.013	0.099	0.749	0.454	−1.232	2.751

Note: *P* < 0.05 meant a statistically significant difference; Knowledge: R2 = 0.108, F = 6.395, *P* <0.001; Attitude: R2 = 0.032, F = 0.927, *P* < 0.001; Practice: R2 = 0.099, F = 5.817, *P* < 0.001. Dummy variables were set for department [Pediatric Emergency Department (0,0), Pediatrics Intensive Care Unit (0,1), and Department of General Pediatrics (1,0)], job position [primary nurse (0,0), nursing manager (0,1), and nursing educator (1,0)], intravenous therapy training (0 for Yes, 1 for No), and nursing hierarchy [CN0 (0,0,0), CN1 (0,0,1), CN2 (0,1,0), and CN3 (1,0,0)]

## Discussion

An on-site investigation that employed the PIVC fixation standard inspection checklist showed that the PIVC fixation pass rate among pediatric nurses was approximately 50%, which is a very low rate. The follow-up questionnaire survey on pediatric nurses’ KAP of PIVC fixation showed that pediatric nurses’ knowledge of PIVC fixation was at a moderate level, while their attitude and practice were at a good level. These patterns were significantly different from the on-site investigation results.

### Status of pediatric nurses’ KAP of PIVC fixation

This study has also shown that pediatric nurses’ knowledge scores on PIVC fixation occur at a moderate level, which is a finding that differs from that of Luo et al., who reported a low level of knowledge of intravenous therapy among the nurses surveyed [15–17]. The reason for this contrast could be that the present study involved pediatric nurses and was limited to PIVC fixation or because the hospital concerned is a teaching hospital and such nurses generally possess more comprehensive knowledge. For attitude, the score was at a good level, which matched the results of Lei’s study on the creation and application of the nurses’ KAP scale for venous access devices [18]. For practice, the scores were also generally good, which reflected that, at the level of the questionnaire, nurses’ fixation practice is good; however, this finding contrasts with the results of the study by Marsh et al., who reported that 69% of PIVCs could not achieve the desired effect due to fixation problems, among other reasons [19]. Nursing managers should minimize the interference of external factors (e.g., skin disinfectant, dressing, and auxiliary fixture) during PIVC fixation [20], provide training on PIVC fixation, and compile standard procedures for PIVC fixation in pediatric patients, thereby improving pediatric nurses’ knowledge and attitude toward PIVC fixation in pediatric patients.

### Factors influencing the KAP of PIVC fixation in pediatric nurses

#### Department

The department in which pediatric nurses are based appears to be a significant factor that influences their scores for knowledge and practice regarding PIVC fixation. The KAP scores of pediatric intensive care unit nurses and the department of general pediatrics nurses were higher than those of pediatric emergency department nurses, which is a finding that contrasts with that reported by Marsh et al. [19], who found that the PIVC failure rate among emergency department patients was lower than that among patients who were waiting to be hospitalized. One possible reason is that PIVCs tend to be implanted in surgical emergency department patients during tense emergency situations, while blood vessels are not impacted by long-term factors. Another reason could be that this study concentrated on both pediatric nurses and PIVC in pediatric patients. Moreover, pediatric emergency department nurses pay more attention to the successful insertion rate and thus avoid conflicts due to insertion failure [21, 22] compared to standard fixation. Overall, PIVC fixation in pediatric patients may be affected by multiple factors, including ward culture, team characteristics, routines, and leadership. Further study on these factors will be required.

#### Job position

Job position appears to be another significant factor that influences pediatric nurses’ knowledge scores, as the knowledge scores of nursing managers were higher than those of primary nurses. Nursing managers are usually head nurses. As nursing quality management representatives, the ability of head nurses to actively acquire knowledge should be better than that of the nurses under their supervision. Head nurses are also nursing educators who are usually responsible for providing training for other nurses. Therefore, it was expected that the head nurses would possess a greater knowledge of PIVC fixation. Receiving systematic training on intravenous therapy techniques can help nurses fix PIVCs with standard methods and significantly reduce the occurrence of iatrogenic complications, thereby improving patient satisfaction and ensuring patient safety [23–25]. Thus, nursing managers should improve the level of knowledge of PIVC fixation among pediatric nurses and create more opportunities for nurses to receive further health education.

#### Nursing hierarchy

Nursing hierarchy appears to be a significant factor that influences pediatric nurses’ practice score, since the scores among CN1 nurses were significantly higher than those of CN0 nurses. CN0 nurses (those with less than 1 year’s length of service) are newly employed nurses who have recently graduated from colleges or finished their standardized training. Inevitably, they lack work experience, and their nursing skills require improvement. Maduemem et al. [26] reported in their study that approximately 37% of nurses feel anxious and nervous when they conduct PIVC puncture and maintenance in children; this feature alone might undermine the puncture and maintenance success rate. First, CN0 nurses should receive more attention and assessment; second, the inspection of PIVC fixation by CN0 pediatric nurses should be conducted to obtain immediate feedback; and third, nurses with more work experience should communicate with CN0 nurses to help them overcome related psychological impediments [27].

### Limitations

This is a short-term study that was conducted within 2 months. The research objects of this study were pediatric nurses and PIVCs in pediatric patients at a maternity and children’s hospital. Both the sample size and diversity were limited, and the results of the multivariate linear regression analysis require further study. We did not consider the factors related to the demographic information of patients and PIVC characteristics, e.g., PIVC-patient ratio, which is also a limitation of this study. The questionnaires were designed, provided, collected, and analyzed by an unblinded hierarchically superior nurse, which could have influenced the participation and responses of the study participants.

This study aimed to investigate the pass rate for PIVC fixation in pediatric patients and factors influencing pediatric nurses’ knowledge, attitude and practice (KAP) concerning peripheral intravenous catheter fixation.

## Conclusion

In the current study, the pass rate of PIVC fixation in pediatric patients was found to be low, while the scores of the practice dimension of the KAP questionnaire on PIVC fixation were found to be high. The inconsistency of these outcomes may lead researchers to further reflect on barriers to the transference or implementation of these recommendations into clinical practice.

Second, PIVC fixation in pediatric patients is affected by multiple factors. In this particular study setting, the KAP level of pediatric nurses regarding PIVC fixation needs to be improved. It is suggested that nursing managers or educators, based on studies of the influencing factors, should carry out interventions and training courses to improve the level of KAP regarding PIVC fixation in pediatric nurses, which should help improve fixation outcomes.

## Data Availability

The datasets generated and/or analyzed during the current study are not publicly available due to the need to maintain the anonymity of participants and the confidentiality of the data. However, the datasets are available from the corresponding author on reasonable request.

## Additonal files

**Additional file 1.** Pediatric PIVC Fixation Standard Checklist (First insertion). Pediatric PIVC Fixation Standard Checklist (Routine maintenance). SOP for Peripheral Intravenous Catheter Fixation. **Additional file 2.** Questionnaire about pediatric nurses’ knowledge, attitude, and practice of PIVC fixation.

## Abbreviations

PIVC: Peripheral intravenous catheter
KAP: Knowledge, attitude, and practice
