# Supplementary material for: Current practices of peripheral intravenous catheter fixation in pediatric patients and factors influencing pediatric nurses’ knowledge, attitude and practice concerning peripheral intravenous catheter fixation: a cross-sectional study
Source: BMC Nurs. 2021 Nov 23;20:236. doi: 10.1186/s12912-021-00758-1 (PMC8609804; doi:10.1186/s12912-021-00758-1)
Supplement: Supplementary file 1 — Additional file 1. Pediatric PIVC Fixation Standard Checklist (First insertion). Pediatric PIVC Fixation Standard Checklist (Routine maintenance). SOP for Peripheral Intravenous Catheter Fixation. [file 12912_2021_758_MOESM1_ESM.doc]

**Pediatric PIVC Fixation Standard Checklist (First insertion)**

|  | Checklist Items | | | | | Result |
| --- | --- | --- | --- | --- | --- | --- |
| BBed no. | A. Did skin dry naturally after performing skin antisepsis? | B. Was dressing fixed with the standard technique | C. Was PIVC joint fixed with Ω technique? | D.Was auxiliary fixtures used correctly? | E.Was health education provided? | Was PIVC fixation rated ‘Pass’? |
| 1 |  |  |  |  |  |  |
| 2 |  |  |  |  |  |  |
| 3 |  |  |  |  |  |  |
| 4 |  |  |  |  |  |  |

**Pediatric PIVC Fixation Standard Checklist ([Routine maintenance](http://dict.youdao.com/w/eng/routine maintenance/?spc=routine maintenance" \l "keyfrom=dict.typo)**)

|  | Checklist Items | | | | | Result |
| --- | --- | --- | --- | --- | --- | --- |
| BBed no. | A. Was dressing fixed with standard technique? | B. Was the dressing replaced in time when it was abnormal? | C. Was PIVC joint fixed with Ω technique? | D. Was auxiliary fixtures used correctly? | E. Was health education provided? | Was PIVC fixation rated ‘Pass’? |
| 1 |  |  |  |  |  |  |
| 2 |  |  |  |  |  |  |
| 3 |  |  |  |  |  |  |
| 4 |  |  |  |  |  |  |

Note:

1. Please insert “√” or “×” in the answer boxes in accordance with the requirements; “√” for Yes, “×” for No, and tick the box in the summary reasons below. If there are other reasons, please provide the reasons. If any checklist item is marked with “×”, insert “×” for Result. PIVC fixation techniques were rated “Pass” only if all 5 questions in the checklist items were marked with “√”.

2. The definition of natural drying, standard technique of dressing fixation, standard technique of indwelling needle joint fixation, correct use of auxiliary fixation device, and definition of abnormal application of dressing are explained in the SOP for Peripheral Intravenous Catheter Fixation; the detailed content of health education is provided.

**SOP for Peripheral Intravenous Catheter Fixation**

1.1 Disinfectant: 0.5% chlorhexidine gluconate (CHG) ethanol solution is recommended for children >2-months-old

1. 2 Natural to stay dry^[1]^

1.3 Disinfection range: 8×8 cm^2^; Disinfected area > dressing area

1.Skin disinfection

2.1 Tension-free placement: Place the dressing on the indwelling needle centered on the puncture point

2.2 Shaping: Based on the shape of the protruding part of the indwelling needle catheter, shape by hand to make the application closely fit the protruding part

2.3 Pressing: The puncture point as the center from the inside to the outside, so that the application and the skin closely bonded^[2]^

2.4 Evaluation: Before each infusion, the application situation should be evaluated. Abnormal application [3] should be immediately replaced before infusion treatment

2. Dressing fixation

3.1 Fixed position: So as not to affect the observation of the puncture point; does not affect the function bit; avoid causing stress injury and nerve compression

3.2 Fixed: PIVC joint extension tube, using medical adhesive in the joints Ω fixed joint is higher than the skin (QS)

3.3 Evaluation: Before each infusion, the PIVC extension tube fixation should be evaluated, and the abnormal extension tube fixation [4] should be re-fixed before the infusion treatment

3. Joint fixation

4.1 Principle of use: Auxiliary fixing device can be used when necessary, and should be removed immediately as soon as the situation permits^[5]^

4.2 Fixed degree: The tightness of the auxiliary fixing device is appropriate^[6]^

4.3 Principles of fixation: Auxiliary fixation devices do not affect the observation of the puncture site

4. Fixation of auxiliary devices

5.1 Individualized and continuous standard fixed health education

5.2 To evaluate the standard fixed health education

5. Education

Note:

1. Natural drying refers to intentionally shortening the drying time without using other means, such as swab wiping and fan.
2. Tightly fitting means that there is no gap between the application and the skin, and it is appropriate for the skin to be slightly wrinkled.
3. Abnormal application refers to the situation of curling, loosening, moisture, pollution, and damage to the integrity of the application.
4. The extension tube fixed exception: Ω fixed tape loose; QS connector causes compression to the skin (crimson and imprint).
5. Permissible condition refers to the condition where infusion speed, blood circulation, and skin integrity are not affected; the completion of single infusion therapy.
6. Appropriate tightness means that it does not affect the infusion speed, i.e., it does not affect the infusion treatment due to the fixed mode; does not affect blood circulation, skin integrity, i.e., the skin should not be indented, pressurized, and show skin lesions; does not affect the placement of joint functional position, and other auxiliary fixation device materials should be soft, skin-friendly, and breathable.
