# Supplementary material for: Current practices of peripheral intravenous catheter fixation in pediatric patients and factors influencing pediatric nurses’ knowledge, attitude and practice concerning peripheral intravenous catheter fixation: a cross-sectional study
Source: BMC Nurs. 2021 Nov 23;20:236. doi: 10.1186/s12912-021-00758-1 (PMC8609804; doi:10.1186/s12912-021-00758-1)
Supplement: Supplementary file 2 — Additional file 2. Questionnaire about pediatric nurses’ knowledge, attitude, and practice of PIVC fixation. [file 12912_2021_758_MOESM2_ESM.doc]

**Questionnaire about pediatric nurses’ knowledge, attitude, and practice of PIVC fixation**

**Part 1 Demographic information**

1. Which department are you working in?
2. Emergency Department
3. Intensive Care Unit
4. General Pediatrics
5. Your years of experience(including standardized training years)
6. <3 years
7. 3-5 years
8. 6-10 years
9. ≥11 years
10. Your current professional title
11. Nurse
12. Nurse practitioner
13. Supervising nurse
14. Your current nursing Hierarchy
15. CN0 (including standardized training)
16. CN1
17. CN2
18. CN3
19. Your current educational background
20. Junior college diploma
21. Undergraduate
22. Postgraduate
23. Your current job situation
24. Primary nurse
25. Nursing manager
26. Nursing educator
27. Have you received any intravenous therapy training
28. Yes
29. No

**Part 2 KAP questionnaire**

1. **Knowledge**

(The following items are related to your knowledge of PIVC fixation. Please judge true or false and answer the questions).

**K1**. In the absence of intravenous treatment, PIVC can be extended to 48 hours for a full evaluation.

1. Right
2. Wrong
3. Don’t know

**K2.** Evaluation of PIVC fixation includes the integrity of the application/fixation device, patient skin condition, degree of comfort, and potential risk of skin injury.

A. Right

B. Wrong

C. Don’t know

**K3.** There is no need to replace the application when the PIVC application is curled and loosened, but it is necessary to replace the application in time when moisture, pollution, and integrity are damaged.

A. Right

B. Wrong

C. Don’t know

**K4.** The skin should be dried naturally after disinfection.

A. Right

B. Wrong

C. Don’t know

**K5.** The fixation of PIVC should not affect the observation of the puncture site, and should not cause blood circulation, pressure injury, or nerve compression.

A. Right

B. Wrong

C. Don’t know

**K6.** The transparent dressing should be pasted around the puncture point

A. Right

B. Wrong

C. Don’t know

**K7.** The correct method of transparent dressing fixation is divided into three steps: pressure sticking, shaping and caressing.

A. Right

B. Wrong

C. Don’t know

**K8.** In order to ensure the completion of treatment, pediatric patients can routinely use auxiliary fixation devices to increase the firmness of catheter fixation.

A. Right

B. Wrong

C. Don’t know

**K9.** Specification of auxiliary fixation devices includes medical tape, elastic bandage, and non-elastic bandage.

A. Right

B. Wrong

C. Don’t know

**K10.** Comprehensive and personalized health education is not required for patients carrying peripheral venous indwelling needles or their family members, only PICC, CVC, and infusion port are required.

1. Right

B. Wrong

C. Don’t know

**A: Attitude**

(The following items are your opinions about the standard PIVC fixation. Please answer the questions).

**A1.** A timely and comprehensive evaluation of PIVC

1. Not important at all
2. Not important
3. Moderately
4. Important
5. Very important

**A2.** Timely replacement of non-standard transparent dressing

A. Not important at all

B. Not important

C. Moderately

D. Important

E. Very important

**A3.** Standard drying time and method after skin disinfection

A. Not important at all

B. Not important

C. Moderately

D. Important

E. Very important

**A4.** Fix in strict accordance with the standard fixing technique of application

A. Not important at all

B. Not important

C. Moderately

D. Important

E. Very important

**A5.** Correct selection and use of auxiliary fixtures according to specifications

A. Not important at all

B. Not important

C. Moderately

D. Important

E. Very important

**A6.** Comprehensive and personalized health education related to standard PIVC fixation

A. Not important at all

B. Not important

C. Moderately

D. Important

E. Very important

**P: Practice**

(The following items are about your clinical nursing work. Please answer the questions according to your actual situation).

**P1.** You will conduct a comprehensive assessment of the patient’s PIVCs, including the integrity of the dressing/fixation, the patient’s skin condition, degree of comfort, and potential risk of skin injury.

1. Never
2. Occasionally
3. Sometimes
4. Often
5. Always

**P2**. When the patient’s dressings appear curling, loose, moist, contaminated, or damaged in integrity, you will change the dressings in time.

A. Never

B. Occasionally

C. Sometimes

D. Often

E. Always

**P3.** After skin disinfection, you are allowed to dry naturally.

A. Never

B. Occasionally

C. Sometimes

D. Often

E. Always

**P4.** When you fixed the PIVC, you did not affect the observation of the puncture point.

A. Never

B. Occasionally

C. Sometimes

D. Often

E. Always

**P5.** You used the technique of “tension-free placement, shaping, and pasting” to fix the PIVC transparent application

A. Never

B. Occasionally

C. Sometimes

D. Often

E. Always

**P6.** When you fix the PIVC, do not affect the infusion speed, blood circulation, and skin integrity (including crimping and imprinting).

A. Never

B. Occasionally

C. Sometimes

D. Often

E. Always

**P7.** You have provided personalized and comprehensive health education to patients or their family members who carry PIVC.

A. Never

B. Occasionally

C. Sometimes

D. Often

E. Always
